# Structural and functional characteristics of xenavidin, the first frog avidin from *Xenopus tropicalis*

**DOI:** 10.1186/1472-6807-9-63

**Published:** 2009-09-29

**Authors:** Juha AE Määttä, Satu H Helppolainen, Vesa P Hytönen, Mark S Johnson, Markku S Kulomaa, Tomi T Airenne, Henri R Nordlund

**Affiliations:** 1 Institute of Medical Technology, Biokatu 6, FI-33014 University of Tampere and Tampere University Hospital, Tampere, Finland; 2 Department of Biochemistry and Pharmacy, Tykistökatu 6A, Åbo Akademi University, FI-20520 Turku, Finland

## Abstract

**Background:**

Avidins are proteins with extraordinarily high ligand-binding affinity, a property which is used in a wide array of life science applications. Even though useful for biotechnology and nanotechnology, the biological function of avidins is not fully understood. Here we structurally and functionally characterise a novel avidin named xenavidin, which is to our knowledge the first reported avidin from a frog.

**Results:**

Xenavidin was identified from an EST sequence database for *Xenopus tropicalis *and produced in insect cells using a baculovirus expression system. The recombinant xenavidin was found to be homotetrameric based on gel filtration analysis. Biacore sensor analysis, fluorescently labelled biotin and radioactive biotin were used to evaluate the biotin-binding properties of xenavidin - it binds biotin with high affinity though less tightly than do chicken avidin and bacterial streptavidin. X-ray crystallography revealed structural conservation around the ligand-binding site, while some of the loop regions have a unique design. The location of structural water molecules at the entrance and/or within the ligand-binding site may have a role in determining the characteristic biotin-binding properties of xenavidin.

**Conclusion:**

The novel data reported here provide information about the biochemically and structurally important determinants of biotin binding. This information may facilitate the discovery of novel tools for biotechnology.

## Background

Avidins are high-affinity biotin-binding proteins found in the eggs of oviparous vertebrates including bird, reptilian and amphibian species [1-5]. In addition to its production in the oviduct and secretion to egg white, avidin is expressed in several other tissues of the chicken during injury and inflammation [6]. Avidins analogous to those found in the earliest diverging tetrapod species have been isolated from a few bacterial species: *Streptomyces avidinii *(streptavidin; [7,8]) *Bradyrhizobium japonicum *(bradavidin; [9]) and *Rhizobium etli *(rhizavidin; [10]).

Avidins are homotetrameric proteins, the only known exception being rhizavidin, which is a homodimer [10]. The avidin subunits consist of eight anti-parallel β-strands, which form a β-barrel structure that has a biotin-binding pocket at the open end of the barrel. Avidins interact extraordinarily tightly with a water-soluble vitamin, D-biotin (K_d _in the range of 10^-13 ^to 10^-15 ^M). This exceptional strength of interaction has not only been utilized in various biochemical and biophysical applications [11], but has also led to the production of a number of genetically modified forms of avidin and streptavidin [12]. The biotin-binding modes of avidins, and the amino acids involved in biotin binding are highly conserved among different species even though the similarity between the primary sequences of avidins is relatively low.

In addition to biotin binding, comparisons of avidins, either extracted directly from natural sources [7,13] or produced as recombinant proteins [9,10,14,15], have revealed many differences in their physicochemical properties, e.g., stability and immunogenicity. Consequently, the detailed characterization of novel avidins has provided valuable information that could be exploited in the development of novel molecular tools and devices. For example, a chimeric avidin containing structural parts from both chicken avidin and avidin related protein 4 (AVR4), has been found to be more stable than either one of the native forms [16].

Although avidins are expressed in several different species, only a few avidins have been thoroughly characterized. Experimentally determined three-dimensional (3D) structures are available e.g. for streptavidin [17], chicken avidin [18,19], avidin related protein 2 (AVR2) [20], AVR4 [21] and biotin-binding protein A [15]. These structures have proved that the overall fold of the avidins is the same, but that the subtle structural differences explain the observed differences in the functional characteristics of avidins.

Here we report, to our knowledge, the first biochemical and structural characterization of an amphibian avidin - xenavidin - a frog avidin from *Xenopus tropicalis*. Being the only diploid species in the *Xenopus *genus with a small genome (1.7 × 10^9 ^bp) and short generation time, *X. tropicalis *has proven invaluable, e.g., for understanding the mechanisms of vertebrate embryonic development and for functional genomics (for reviews, see [22,23]). The 56% amino acid sequence identity shared between avidin and xenavidin is relatively high and suggests that avidin and xenavidin have similar biochemical and structural properties. However, like for other avidins such as streptavidin, bradavidin and rhizavidin, in comparison with chicken avidin, xenavidin has it own unique features determined by the subtle changes found in its sequence and structure. Thus, the characterization of xenavidin gives insights into the biochemical and structural determinants of a novel member of the avidin protein family, a family of extraordinary biotin-binders with many prospects for exploitation in biotechnology and nanotechnology.

## Results

### Cloning, expression and purification

Two DNA sequences encoding an avidin-like protein from a frog were originally identified by searching an EST sequence database [24]; after discovering the avidin-like gene from *Xenopus tropicalis*, the avidin gene for *Xenopus laevis*, another frog species, was also identified. Here, we focus on the protein from *Xenopus tropicalis*, which we named xenavidin. The cDNA of xenavidin was cloned into the pFastBac1 baculovirus expression system entry vector, and recombinant xenavidin was successfully produced in Sf9 insect cells using the Bac-to-Bac^® ^baculovirus expression system (Invitrogen). The protein was purified with 2-iminobiotin SepharoseTM (Affiland, Liège, Belgium) affinity chromatography. About 2-3 mg of pure protein was typically obtained per one litre of culture media. Attempts to produce xenavidin in a bacterial expression system in *E. coli *[25] were unsuccessful.

### X-ray structure

**Figure 1:**
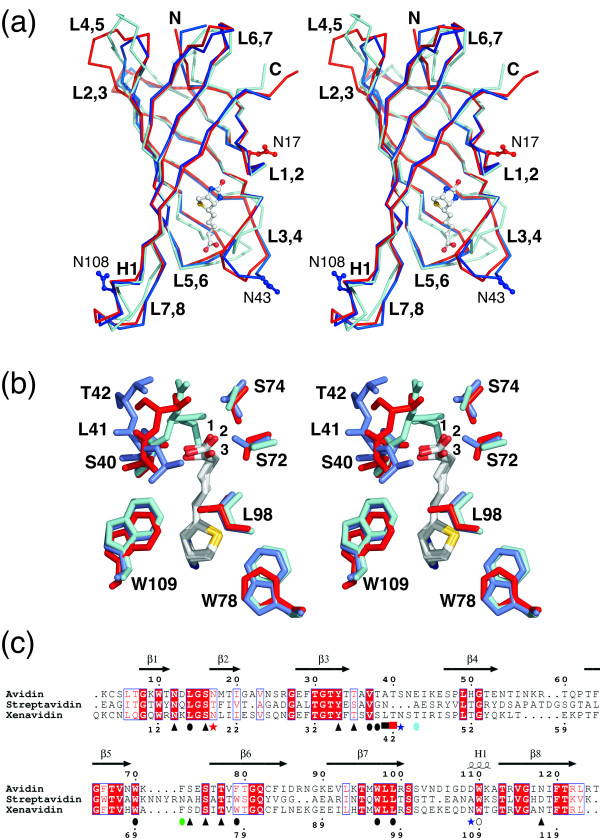
**Comparison of avidin, streptavidin and xenavidin**. A, Structural alignment of avidin [PDB: 1AVD] (red), streptavidin [PDB: 1MK5] (cyan) and xenavidin (reported here; [PDB: 2UYW] (blue)). The A subunits of each protein are shown as ribbons. The bound biotin of xenavidin is shown as sticks. Loops connecting β-strands (for example, L1,2 connecting strands β1 and β2) as well as the amino (N) and carboxyl (C) termini are labelled. The α1-helix (H1) is also indicated. B, Selected residues around the biotin-binding pocket are shown as sticks and are labelled according to xenavidin. Biotin molecules are shown (1, xenavidin; 2 avidin; and 3 streptavidin). C, Structure-based sequence alignment created based on the ensemble of aligned structures shown in A. The secondary structure elements of avidin (β-strands 1-8, arrows; α-helix, H1) are indicated, and residues are numbered according to avidin (top) and xenavidin (bottom). Identical (red background) and physicochemically similar amino acid residues (red letters) are boxed. Potential N-glycosylation sites are indicated with an asterisk. Residues hydrogen bonded to biotin are indicated by triangles (side-chain interaction) or squares (main-chain interaction). Spheres denote residues involved in hydrophobic effect or van der Waals interactions with biotin. Interacting residues from a neighbouring subunit are indicated with an open circle. Colouring scheme for the symbols: black, conserved in all three proteins; green, conserved in xenavidin and avidin only; blue, unique to xenavidin; red, unique to avidin; and cyan, unique to streptavidin. For additional information, see Methods.

**Figure 2:**
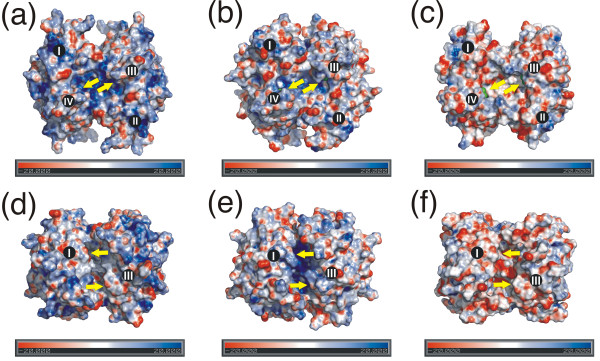
**Molecular surfaces of chicken avidin (A and D; **[PDB: 1AVD]**), xenavidin (B and E; **[PDB: 2UYW]**) and streptavidin (C and F; **[PDB: 1MK5]). The surfaces were colored by electro potentials. The views in A, B and C are rotated 90 degrees around the x-axis in D, E and F, respectively. The subunits (I-IV) are numbered according to Livnah *et al*. [18]. The yellow arrows pinpoint the entry sites for biotin-binding pockets (A-C) and water channels (D-F). The valeric acid moiety end of bound biotin molecules (green spheres) is seen in C.

Xenavidin overexpressed in insect cells was crystallized with and without D-biotin (BTN) added to the crystallization solution. The X-ray structures of the xenavidin-BTN complex [PDB: 2UYW] and the unliganded xenavidin protein [PDB: 2UZ2] were solved to 1.7 Å resolution. The structures crystallized in a rhombohedral space group and had a dimer in their asymmetric unit (for the structure determination statistics, see Table 1). In general, both of the xenavidin structures were highly similar to the known structures of avidin and streptavidin: the 3D structure of xenavidin is homotetrameric, each subunit consisting of an eight-stranded β-barrel (Figure 1). The amino acids lining the biotin-binding pocket are structurally conserved between xenavidin and chicken avidin. There were, however, differences in the loop architecture between these proteins, particularly in the L3,4-loop and the L4,5-loop. The unique L3,4-loop design in different avidins is a key feature regulating the binding affinity of avidins [18,26-28], but the role of the variable L4,5-loop is not clear though it is known not to be directly involved in ligand binding. Moreover, there were some differences in the interface architecture of xenavidin in comparison to chicken avidin. For example, out of the three residues at the 1-3 subunit interface, two (M96 and V115; numbering according to avidin [18]) are conserved between avidin and xenavidin, whereas I117 in avidin is substituted by alanine in xenavidin, probably contributing to the reduced stability of xenavidin (see below). The surface properties of avidin, xenavidin and streptavidin vary, too (Figure 2).

**Table 1:** X-ray structure determination statistics for xenavidin

**Data collection**^**a**^	**Unliganded protein** [PDB: 2UZ2]	**BTN complex** [PDB: 2UYW]
Wavelength (Å)	0.900	0.900
Beamline	ID29 (ESRF)	ID29 (ESRF)
Detector	ADSC	ADSC
Resolution (Å)	25 - 1.7 (1.8 - 1.7)	25 - 1.7 (1.8 - 1.7)
Unique observations	37060 (5778)	37484 (5853)
I/sigma	22.4 (4.7)	22.0 (4.2)
*R*_factor _(%)^b^	6.7 (43.9)	7.4 (50.5)
Completeness	99.9 (100)	99.9 (100)
Redundancy	8.5 (8.6)	9.1 (9.2)
**Refinement**		
Space group	*R*32	*R*32
Unit cell:		
a, b, c (Å)	110.8, 110.8, 142.3	110.9, 110.9, 143.8
α, β, γ (°)	90, 90, 120	90, 90, 120
Monomers (asymmetric unit)	2	2
Resolution (Å)	25 - 1.7	25 - 1.7
*R*_work _(%)^c^	18.1	16.5
*R*_free _(%)^c^	21.6	19.0
Protein atoms	1997	1953
Heterogen atoms	20	62
Solvent atoms	208	224
*R.m.s.d:*		
Bond lengths (Å)	0.014	0.017
Bond angles (°)	1.5	1.5

^a^The numbers in parenthesis refer to the highest resolution bin.

^b^Observed *R*-factor from XDS [55].

^c^From Refmac 5 [60] using TLS [62] & restrained refinement.

**Figure 3:**
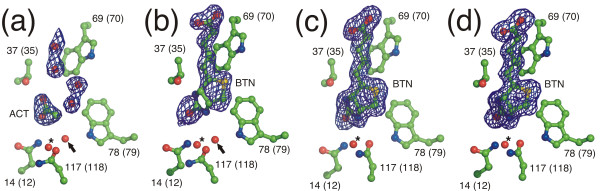
**Ligand binding to xenavidin**. Simplified presentation of the ligand-binding sites: A, subunit A of unliganded xenavidin; B, subunit D of unliganded xenavidin; C, subunit A of the xenavidin-BTN complex; and D, subunit D of the xenavidin-BTN complex. Amino acid residues are numbered according to xenavidin and avidin (in brackets). Colouring scheme for the stick models: red, oxygen atoms; blue, nitrogen atoms; green, carbon atoms. Water molecules are shown as red spheres. ACT, acetate ion; BTN, D-biotin. Difference Fo-Fc electron density map (blue) contoured at 3σ around the bound ligands is depicted; the maps were calculated in the absence of ligands. An asterisk indicates the conserved water molecule found at the entrance of the water channel, at site 2 [33], and an arrow pinpoints the water molecule at site 1 [33] that was present only in the unliganded xenavidin structure (see text for details).

The subunits of the dimer found in the asymmetric units of the xenavidin crystals are labelled A and D to match the numbering system generally used to describe the biological unit of chicken avidin (tetramer; subunits I-IV; [18]). In the unliganded structure of xenavidin, there was no clear electron density for biotin in subunit A. However, weak electron density was evident for the ligand at the binding site in subunit D; thus, a biotin molecule was built into subunit D of the unliganded xenavidin structure despite the presence of weak electron density around the ligand (Figure 3). One possible explanation for the varying electron densities at the biotin-binding sites could be sequential binding, which is supported by some previous studies suggesting cooperativity between binding sites of (strept)avidin [29,30]. However, some other studies do not support cooperativity between binding sites [31,32]. In the BTN-complex structure, D-biotin was clearly present in both of the biotin-binding sites of the asymmetric unit. In the X-ray structures of xenavidin there was no clear evidence that either of the two potential N-glycosylation sites of each polypeptide, N43 or N108 (S41 and D109 in avidin; Figure 1), would in fact be glycosylated. However, the presence or absence of glycosylation at N43 cannot be reliably established since N43 is within the L3,4-loop and the side-chain atoms of this residue have high B-factors and hence poor electron density. The structures of xenavidin clearly showed that N108 did not have sugar units attached to its side chain.

In xenavidin, three amino acids residues out of 18 lining the biotin-binding pocket are different from those in the binding pocket of avidin: the equivalent residues for the non-conserved S40, L41 and W78 found in xenavidin in avidin are T38, A39 and F79 (Figure 1). S40/T38 and W78/F79 interact with biotin through hydrophobic effect and van der Waals interactions, whereas L41/A39 are involved in hydrogen bonding via the residue's main-chain nitrogen atom. In addition, the main chain of T42 in xenavidin is not close enough to form a hydrogen bond with biotin ([PDB: 2UYW], the closest approach distance is over 4 Å), whereas in avidin a hydrogen bond between the main-chain nitrogen atom of the equivalent residue T40 is evident ([PDB: 1AVD], distance = 3 Å; [19]). These subtle differences in the architecture of the biotin-binding sites of xenavidin and avidin may contribute, at least partially, to the different biotin-binding properties that were observed for these two proteins (see below).

A water molecule was detected in the unliganded structure of xenavidin at the bottom of the ligand-binding site bridging the carboxamide group of N117 and biotin (bridging was evident only in subunit D; Figure 3); the conformation of N117 in the unliganded structure differs from that in the structure of the xenavidin-biotin complex. To our knowledge, the only other known avidin structures harbouring a water molecule at the corresponding location, referred to as 'site 1' by Hyre *et al*. [33], are two mutant structures of streptavidin: the D128A mutant structure ([PDB: 1SWT]; [34]) and the S45A/D128A double mutant structure ([PDB: 1MEP]; [35]). A structural water at site 1 has so far not been observed in native avidin structures. A conserved water molecule, the water molecule at site 2 ([33]; Figure 3), located at the entrance of the water channel, is present in both of the xenavidin structures. According to Hyre *et al*. [33], biotin dissociation from streptavidin is initiated by penetration of water molecules into these sites. The presence of structural water molecules at site 1 and/or 2 in the xenavidin structures suggests a role for these water molecules in the biotin association/dissociation events of xenavidin; the biotin-binding site of xenavidin may be more vulnerable to potential 'attack' by water molecules than it is in chicken avidin. This hypothesis is supported by our experimental results, which showed an increased dissociation rate of biotin from xenavidin compared to avidin (see below).

### Biochemical and physicochemical characterization

**Figure 4:**
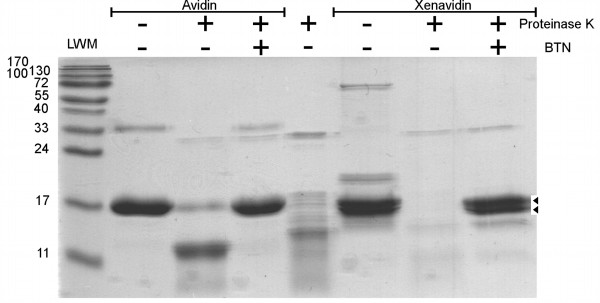
**Limited proteolysis of xenavidin and avidin by proteinase K**. Samples from left to right: LWM, a molecular weight standard (weights in kDa, Fermentas Life Sciences); Avidin control sample; Avidin treated with proteinase K in the absence of biotin; Avidin treated with proteinase K in the presence of biotin; Proteinase K control sample (four times higher concentration of proteinase as compared to the other samples); Xenavidin control sample; Xenavidin treated with Proteinase K in the absence of biotin; Xenavidin treated with proteinase K in the presence of biotin. Black arrowheads indicate the putative single- (lower arrow) and double-glycosylated (upper arrow) xenavidin forms.

The quality and molecular size of the isolated xenavidin protein was analyzed using 15% SDS-PAGE and size exclusion chromatography. In the latter analysis, xenavidin appeared mainly as a single peak and had a size of approximately 74 kDa suggesting a tetrameric quaternary structure (Table 2; [Additional file 1, Additional file 2]). Recall that xenavidin has two asparagine residues, N43 and N108 (Figure 1), identified as potential glycosylation sites according to the program NetNGlyc 1.0 (; Gupta et al. in preparation). The theoretical molecular weight of xenavidin, calculated on the basis of the primary amino acid sequence, is only 60 kDa, which suggests that N43 might indeed be glycosylated in the insect cells. SDS-PAGE analysis resulted in multiple bands where, in addition to the band corresponding to the nonglycosylated form, two higher molecular weight forms of the monomeric protein were detectable. These higher molecular weight forms most probably correspond to glycosylated species with a varying number and type of sugar units (marked with black triangles in Figure 4). It is well known that proteins overexpressed using the baculovirus expression system may have heterogeneous glycosylation patterns, as reviewed by Davies [36]. Furthermore, we have also observed this phenomenon in the AVR-proteins, which may have multiple glycosylation sites per polypeptide chain [37]. Further evidence for the presence of heterogeneously glycosylated xenavidin was obtained by treating the purified protein with the deglycosylation enzymes Endo Hf and PNGase, which caused the disappearance of the higher molecular weight forms of xenavidin in SDS-PAGE analysis (not shown). Moreover, based on the X-ray analysis of xenavidin (see above), the crystallized proteins may have carbohydrates attached to one of the two potential glycosylation sites. Taken together, our data support a structure in which xenavidin is glycosylated at N43; interestingly, the glycosylation site in xenavidin is located at a position equivalent to that glycosylated in AVR4 (N43) [14]. Avidin also has one glycosylation site (N17) but there is no equivalent glycosylation site in xenavidin.

**Table 2:** Gel filtration analysis of xenavidin and avidin. The samples were in the presence (+BTN) or absence (-BTN) of biotin.

**Protein**	**Observed molecular weight (kDa)**	**Theoretical molecular weight^**b **^(kDa)**
Xenavidin (-BTN)	74.5^a^	59.5
Xenavidin (+BTN)	73.3^a^	59.7
Avidin (-BTN)	62.5^a^	57.4

^a^The analyzed proteins were most probably glycosylated; sugar moieties comprise at least 10% of the mass of avidin [69].

^b^Theoretical values were calculated from the primary structures.

**Figure 5:**
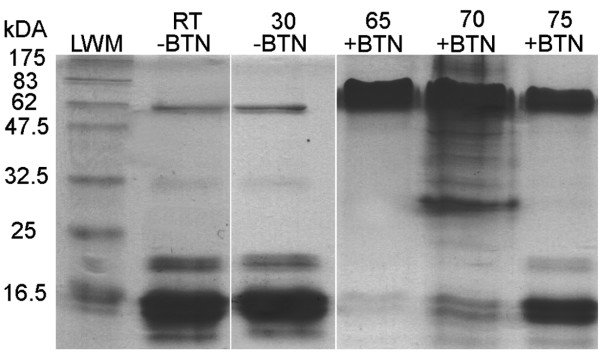
**Thermal stability of the tetrameric forms of xenavidin**. SDS-PAGE -based analysis of xenavidin either in the absence of added biotin (-BTN) or saturated with biotin (+BTN) prior to analysis. The measurement temperatures, 22 (RT, room temperature), 30, 65, 70 and 75°C, are shown, and the molecular weights of the standard proteins (LWM; BioLabs) are indicated (kDa). This figure was created from the images of two different gels; the uninformative parts of the gel images are not shown.

Like almost all of the other avidin-like proteins studied so far, xenavidin also tends to form oligomers of tetramers, which could be seen as a minor peak, with an apparent molecular weight of 210 kDa, before the main peak in the gel filtration chromatogram [Additional file 1, Additional file 2]. These oligomers could not be detected in an SDS-PAGE-based thermostability assay (Figure 5.), where xenavidin appeared only as a tetrameric form in the presence of biotin to up to 65°C, and even at 90°C (not shown), but dissociated into monomers in the absence of biotin already at room temperature (22°C).

Xenavidin has a very similar theoretical isoelectric point (pI 9.23) to avidin (pI 9.69); values for both proteins were calculated using the Compute pI/Mw tool [38]. Hence, xenavidin and avidin are positively charged at neutral pH, whereas streptavidin (pI 6.10) has a negative net charge at the same pH.

Sera from human cancer patients [13] who were exposed to avidin and streptavidin were used to probe the cross reactivity among xenavidin, chicken avidin and streptavidin. In this analysis xenavidin did not react significantly with the avidin/streptavidin immunized sera. Moreover, polyclonal antibodies against avidin and streptavidin did not recognize xenavidin. Taken together, xenavidin appears not to be immunologically cross reactive with (strept)avidin [see Additional file 3]. The complete dataset is reported in Helppolainen *et al*. [39].

### Protein stability

The thermal stability of xenavidin was determined with a SDS-PAGE thermostability assay (Figure 5), and the analysis was performed as described by Bayer *et al*. [40]. Without biotin, the tetrameric form of xenavidin was unstable and the protein was monomeric already at room temperature (22°C). When biotin was added, the stability of the tetrameric form of the xenavidin was significantly increased - the transition temperature of dissociation of the tetramer was raised to 75°C. Therefore, in the absence of biotin, the tetrameric quaternary structure of xenavidin is clearly more vulnerable to the denaturing effect of SDS than is the structure of avidin [40], whereas in the presence of biotin, xenavidin and avidin have relatively similar stabilities, thus suggesting high affinity to biotin.

The stability of xenavidin against proteinase K was tested in the presence and absence of biotin. As previously shown, avidin was cleaved in the absence of biotin after extended incubation with proteinase K to fragments of 10 kDa and below, but the presence of biotin protected avidin from proteolytic cleavage [41]. Similar behaviour was observed in the case of xenavidin. In the absence of biotin, xenavidin was cleaved by proteinase K into small fragments, which could not be seen using gel analysis, whereas adding biotin to the reaction mixture prevented cleavage (Figure 4).

### Ligand binding properties

**Figure 6:**
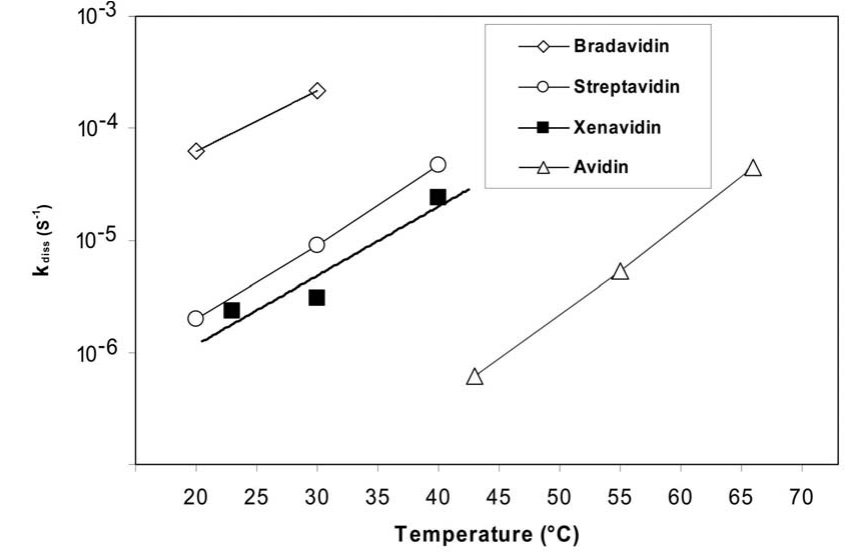
**[^3^H]biotin dissociation analysis**. The dissociation rate constant of *d*-[8,9-^3^H]biotin was measured at different temperatures. The values for bradavidin, streptavidin and avidin are from references [9,63] and [14], respectively.

**Figure 7:**
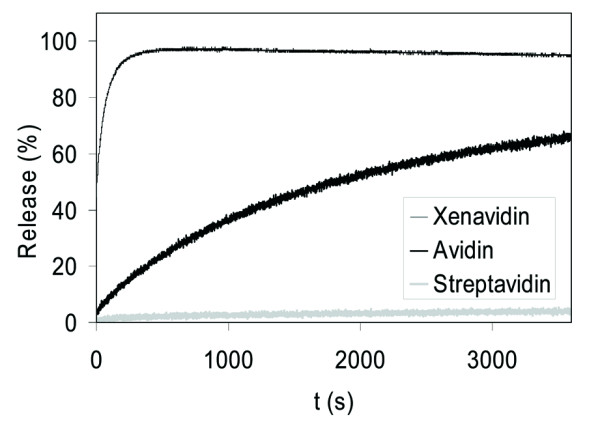
**Dissociation analysis of fluorescent biotin**. The dissociation of Bf560-labelled biotin was measured at 50°C.

The dissociation rate of d-[8,9-3H]biotin from xenavidin samples was measured at several different temperatures (Figure 6). Xenavidin showed a faster dissociation rate compared to avidin, but a slower rate when compared to either streptavidin or bradavidin ([9]; Figure 6). If a similar biotin-association rate constant were assumed for all the proteins, xenavidin would have a streptavidin-like affinity for biotin (K_d _= 1 × 10^-13 ^M). The dissociation of biotin from xenavidin was also measured by fluorescence spectroscopy using fluorescently labelled Bf560-biotin [25]. Xenavidin released this biotin conjugate much faster than did avidin - again suggesting a weaker affinity for biotin (Figure 7; Table 3). It is important to keep in mind that avidins may differ in the way in which they bind different biotin forms, including free biotin (d-[8,9,3] biotin) and conjugated biotin (Bf560-biotin) [42]. For example, it has been shown that streptavidin binds Bf560-biotin more tightly than avidin, whereas avidin binds free biotin better than streptavidin [9]. In our opinion, the fluorescent biotin assay reflects more the conditions found in life science applications, where biotin is typically conjugated to other molecules, whereas the free biotin assay mimics more closely the conditions *in vivo*. The determination of the kinetics of association of free biotin would give an even deeper picture of the ligand-binding properties of avidins and would be interesting to do in future experiments.

**Table 3:** Dissociation analysis of fluorescent Bf560-biotin

**Protein**	**Dissociation rate constant at 25°C** **s^**-1 **^(× 10^**-5**^)**	**Dissociation rate constant at 50°C** **s^**-1 **^(× 10^**-5**^)**	**Release after 1 h at 50°C** **%**
Xenavidin	17.0	442.1	95.5
Avidin	2.3	32.6	76.5
Streptavidin	--^a^	0.7	5.1

^a^The dissociation rate was too slow to be measured accurately at 25°C.

The ligand-binding characteristics of xenavidin were also studied using a surface plasmon resonance approach using an optical biosensor instrument (Biacore X, GE Healthcare). In this analysis, xenavidin showed ~10-fold lower affinity (K_d _= 1 × 10^-6 ^M) to the 2-iminobiotin functionalized optical biosensor chip compared to chicken avidin (K_d _= 1 × 10^-7 ^M). Both a slower association rate and a faster dissociation rate were observed for xenavidin compared to chicken avidin (Table 4).

**Table 4:** Optical biosensor analysis (Biacore X) of 2-iminobiotin binding

**Protein**	**k_**ass**_(s^**-1**^) (× 10^**4**^)**	**k_**diss **_(M^**-1**^s^**-1**^) (× 10^**-3**^)**	**K_**d **_(M) (× 10^**-8**^)**
Xenavidin	2.2	21.6	99.8
Streptavidin	20.0	17.0	8.5
Avidin	9.4	9.6	10.2

## Discussion

Although thoroughly studied in terms of structural, biochemical and biophysical characteristics, the biological functions of avidins are not fully understood. Here, we expand the research on avidins by reporting xenavidin, the first avidin-like protein from a frog, which was identified as an expressed sequence tag (EST) from the *Xenopus tropicalis *genome project. Avidins appear to be conserved among egg-laying vertebrates [3,4,6] and are thought to have a role as defence proteins against microbial infections [43,44]. Bird eggs contain egg yolk, the compartment for embryonic development, and the egg white that surrounds the egg yolk provides both nutrients and protection for the embryo. In frogs, the equivalent to egg white is called egg jelly [45]. The avidin content of egg jelly may have an important role in the embryonic development of frogs, a hypothesis that is also supported by recent studies within natural bird populations, in which the concentration of avidin in egg white appears to be linked to hatching success (Siitari *et al*. manuscript in preparation). In this light, it seems likely that avidins are found in all frog and bird proteomes. It would be interesting to see whether some of the many fish species also carry avidin in their proteome; a search of sequence databases revealed that the zebrafish (*Danio rerio*), for example, may carry a gene encoding an avidin-like protein (data not shown). The first avidin from fungi, tamavidin, has recently been reported, too [46].

Avidin and streptavidin have been used for pre-targeting immunotherapy and imaging [47,48]. Although the folds of these proteins are similar, and both bind biotin tightly, they differ in their pharmacokinetic characteristics [49,50]. The glycosylation of avidin, for example, was previously observed to affect its uptake by tumours and accumulation in the liver. On the other hand, the basic pI of avidin is responsible for its accumulation in the kidneys [51,52]. Therefore, avidin cannot be efficiently used in drug delivery and imaging owing to non-specific binding to extraneous material [53]. In comparison to avidin, streptavidin is known to have a longer half-life in plasma, but it accumulates mainly in the kidneys [49]. Since xenavidin has unique pharmacokinetic properties, for example its glycosylation pattern and electropotential surface properties (data not shown), it could be a valuable substitute for avidin or streptavidin in biomedical applications.

The X-ray structures of xenavidin show that xenavidin has a β-barrel fold like the other known structures of avidins. The residues around the biotin-binding pocket are also well conserved, but some differences were still found - four residues of xenavidin (S40, L41, T42 and W78) had altered interactions with biotin when compared to the equivalent residues of avidin (T38, A39, T40 and F79), the alterations affecting hydrogen bonding via the main chain, hydrophobic effect or van der Waals interactions (Figure 1). S40, L41 and T42 are located within the L3,4-loop, which is known to be important for determining the ligand-binding specificity of avidins [18,27,28]. Although T42 in avidin is comparable to T40 in xenavidin, the interaction with biotin is formed in a different way. Together with the unique subunit interface design, these, even subtle structural changes may be responsible for the observed, unique ligand-binding properties of xenavidin. Moreover, in the unliganded xenavidin structure, a water molecule was found at a site referred to as site 1 [33], located at the deep end of the biotin-binding pocket (indicated with an arrow in Figure 3). In the biotin-complex structure of xenavidin, this water molecule was missing, even though a conserved water molecule at the entrance of the water channel was observed in both the unliganded protein structure and the biotin-complex structure (this site is referred to as site 2 by Hyre *et al*. [33]; indicated with an asterix in Figure 3). Thus, the unliganded structure of xenavidin may represent the first example of an intermediate structure of the avidin family, with a structural water molecule located deep within the biotin-binding pocket and lending support to the water-bridged model of biotin association/dissociation [35]. Moreover, Freitag *et al*. [34] and Hyre *et al*. [35] have shown that D128 in streptavidin has an important role in controlling the movement of water molecules in this area. Interestingly, N117, the residue analogous to D128 of streptavidin and N118 of avidin, has an altered conformation in unliganded xenavidin compared to N117 of the biotin-complex structure of xenavidin, suggesting a potentially important role for this residue in the water-mediated association/dissociation mechanism of avidins in general.

## Conclusion

We have carried out a structural and functional analysis of xenavidin, a novel avidin from *Xenopus tropicalis*. Our results show that xenavidin is a well-conserved member of the avidin protein family having a tetrameric structure, N-glycosylation and extremely tight biotin-binding capability that is mediated via numerous non-covalent interactions including hydrogen bonds, hydrophobic interactions and van der Waals contacts. However, biochemical and structural features unique for xenavidin were also discovered. Our results may facilitate the development of novel and advanced avidins for biotechnological and nanotechnological applications. Overall, this study deepens our understanding of the structure-function relationship of avidins.

## Methods

### Cloning

The cDNA of xenavidin was amplified by PCR using an EST clone of *Xenopus tropicalis *([clone ID: AM14731C6], Geneservice Ltd, ), [GenBank: CF523241] as a template, Xen_5' as a forward primer (5'-GAGAC**AGATCT**ATGAACGCTCTCACTCTCCT-3') and Xen_3' as a reverse primer (5'-CACAG**AAGCTT**TTATTCCTTGCGGATTTTTT-3'). The primers had restriction enzyme sites for *Bgl*II (forward primer) and *Hind*III (reverse primer), which are underlined. After isolation from a preparative agarose gel, the PCR product was digested with *Bgl*II and *Hind*III (Fermentas Life Sciences) and cloned into the pFastBac1 baculovirus expression system entry vector, which had been treated with the *Bam*HI and *Hind*III restriction enzymes (Fermentas Life Sciences). The resulting expression construct (pFastBac1-xenavidin) was confirmed by DNA sequencing.

### Expression and purification

Xenavidin was produced as a soluble recombinant protein using the Bac-To-Bac^® ^baculovirus expression system (Invitrogen/Finnzymes, Espoo, Finland). Recombinant baculovirus genomes were generated by transforming *E. coli *DH10Bac cells (Invitrogen/Finnzymes, Espoo, Finland) with the pFastBac1-xenavidin vector. The isolated recombinant baculoviruses were then used to transfect *Spodoptera frugiperda *Sf9 insect cells by lipofection using the Cellfectin^® ^reagent according to the instructions of the manufacturer (Invitrogen Corporation, Carlsbad, CA). The virus stock was amplified by infecting a suspension culture of insect cells and then used to produce xenavidin in insect cells transferred to biotin-free medium. Xenavidin was purified from insect cells using 2-iminobiotin affinity chromatography as reported earlier in detail by K. Airenne *et al*. [54].

### Crystallization and X-ray structure determination

Suitable conditions for crystallization of xenavidin were initially investigated at 22°C using the Classics™ (Nextal Biotechnology) and Wizard II (Emerald Biostructures) screens, the vapour diffusion method and sitting drops (1.3-2 μl) on 96 well plates (Corning Inc.). The protein solution (~1.5 mg/ml) contained 50 mM sodium acetate (pH 4) and 20 mM sodium chloride. In order to prepare xenavidin - biotin complexes, the protein solution was mixed with a biotin (Sigma) solution (1 mg/ml) containing 5 mM Tris (pH 8.8) and 8 mM CHES (pH 9.5) using a 10:1 (v/v) ratio of the protein and ligand solutions, respectively. Three crystals, which were used to solve the X-ray structures of xenavidin, formed in the following drop conditions: crystal-1 (xenavidin-biotin complex), 1 μl of protein solution with biotin and 0.7 μl of well solution (10% (w/v) PEG 1000, 10% (w/v) PEG 8000); crystal-2 (xenavidin-biotin complex), 0.5 μl of protein solution with biotin and 0.7 μl of well solution (5% (v/v) isopropanol, 2.0 M ammonium sulfate); and crystal-3 (unliganded xenavidin), 0.6 μl of protein solution without biotin and 0.7 μl of well solution (1.0 M sodium citrate, 0.1 M Tris (pH 7), 0.2 M sodium chloride). These rhombohedral crystals formed in a few days or weeks and had dimensions typically smaller than 0.1 × 0.1 × 0.1 mm.

The first X-ray diffraction data set of the xenavidin-biotin complex was collected from crystal-1 at the MAX-lab beam line I911_2 (Lund, Sweden) using a MarCCD detector and a second data set from crystal-2 at the ESRF beam line ID-29 (Grenoble, France) using an ADSC detector, both at 100 K. The beam line ID-29 was also used to collect data from the unliganded structure of xenavidin (crystal-3). The crystals were cryoprotected by adding either 0.7 μl (crystal-1) or 1.0 μl (crystal-2 and -3) of 4 M sodium formate to the crystallization drop just prior to flash-freezing in a 100 K liquid nitrogen stream (Oxford Cryosystem). The diffraction data were processed to 1.9 Å (crystal-1; data not shown) and 1.7 Å resolution (crystal-2 and -3; Table 1) using the XDS program package [55]. The 1.9 Å data were used for the initial structure determination of the xenavidin-biotin complex, which was carried out using the molecular replacement program Phaser [56] of the CCP4i program suite [57,58] and a dimeric polypeptide model of AVR2 (1WBI; [20]) as a search model. The initial 1.9 Å model was rebuilt using ARP/wARP (starting from an existing model; v. 6.1.1) [59], refined with Refmac5 [60] and modified with Coot [61]. Solvent atoms and other non-protein atoms were added to the model either with the automatic procedure of ARP/wARP or Coot, or manually in Coot. The 1.7 Å data (crystal-2) were used to extend the resolution of the pre-existing 1.9 Å model and to build the final model of the xenavidin-biotin complex - the 1.9 Å model was used as a starting model in ARP/wARP and the rebuilding steps described above were followed with the exception that TLS option [62] was used in the restrained refinement (Refmac5). The structure determination for the unliganded structure of xenavidin was carried out as described for the 1.7 Å xenavidin-biotin complex structure.

The final 1.7 Å unliganded xenavidin and xenavidin-biotin complex structures were analyzed using the inbuilt tools of Coot before deposition to the Protein Data Bank with entry codes 2UZ2 and 2UYW, respectively. The data collection and structure determination statistics are summarized in Table 1.

### Optical biosensor analysis

The Biacore X optical biosensor instrument was used in the analysis, where the CM5 sensor chip was functionalized with 2-iminobiotin. The functionalization of the chip was done in three phases: First, the carboxymethyl dextrane was activated using a mixture containing 0.2 M EDC (1-ethyl-3-(3-dimethylaminopropyl) carbodiimide hydrochloride) and 0.05 M NHS (N-hydroxysuccinimide) in water. Second, in order to introduce amino groups to the surface, 1 M ethylenediamine (Fluka 03550) in water was applied. Third, a mixture of EDC (0.2 M) and 2-iminobiotin (10 mM) was applied on the surface in 25 mM Na-phosphate pH 6.5. The amount of 2-iminobiotin attached on the surface was 81 response units.

Xenavidin was injected onto the sensor chip in a 50 mM sodium carbonate buffer (pH 11.0) containing 1 M NaCl at 25°C and using a flow rate of 50 μl/min. Chicken avidin expressed in *E. coli *[25] or streptavidin from *Streptomyces avidinii *(Biomol GmbH, Hamburg, Germany) were analyzed as control samples; the control samples incubated with an excess of biotin showed no binding to the surface. The surface was regenerated between measurement cycles by injecting 20 μl of 0.5 M acetic acid followed by washing with the measurement buffer.

BIAevaluation software v. 4.1 was used for data analysis, and a global fitting method was used to analyze the data set. A period of 20 seconds from the beginning of each injection was included in the association phase fit and the dissociation rate analysis was performed for a 60-second period starting immediately after the end of the injection.

### Radioactive biotin dissociation assay

The dissociation rate constant of *d*-[8,9-^3^H]biotin (Amersham, Buckinghamshire, England) from avidin and xenavidin was determined at different temperatures by competition with free biotin as described in Klumb *et al*. [63]. Measurements were carried out in neutral pH in a 50 mM sodium phosphate buffer containing 100 mM NaCl and 10 μg/ml of BSA to prevent non-specific binding. After the measurement of free radioactive biotin, xenavidin was added to a concentration of 50 nM. The level of unbound radioactive biotin was measured and an excess of normal biotin was added to the samples to replace bound radioactive biotin. Free biotin was separated from the bounded one by centrifugation through Microcon^® ^30 kDa molecular weight cut off centrifugal filter devices (Millipore). The fractions of biotin bound at each time point were used to create a plot of ln(fraction biotin bound) versus time. The dissociation rate constant (k_diss_) is determined from the slope of the best-fit line:

(1)

where *x*_*t *_is the total amount of ligand before the addition of protein, *x *is the free biotin concentration at each time point and *x*_0 _is the amount of free ligand in the presence of protein just prior to the addition of nonradioactive biotin.

### Fluorescence spectroscopy

The Quanta Master™ spectrofluorometer (Photon Technology International Inc., Lawrenceville, NJ) with a PTI Fluorescence Master System and the software Felix 32 was used to measure the biotin-binding properties of avidin, xenavidin and streptavidin. The analyses were done in a 50 mM sodium phosphate buffer (pH 7.0) containing 650 mM NaCl at two different temperatures (25 ± 1°C and 50 ± 1°C) by measuring the reversing of quenching of the biotin-coupled fluorescent probe ArcDia™ Bf560 (ArcDia Ltd., Turku, Finland) due to competition with free biotin (100-fold molar excess) as previously described [25]. The spectrum of Bf560-biotin in the presence and absence of avidin is shown in [Additional file 4].

### Gel filtration

Gel filtration analysis was performed for xenavidin, avidin and streptavidin using an ÄKTA™ purifier HPLC instrument (Amersham Biosciences AB, Uppsala, Sweden) equipped with a Superdex 200 10/300 GL column (Tricorn, Amersham Biosciences AB, Uppsala, Sweden) as previously described [15]. The analyses were performed in a 50 mM sodium phosphate buffer (pH 7.0) containing 650 mM NaCl. Protein-biotin complexes were prepared by incubating each sample in an excess of biotin for 10-20 minutes at room temperature (22°C) prior to analysis.

### SDS-PAGE thermostability assay

SDS-PAGE-based stability analysis was performed for xenavidin and avidin as described by Bayer *et al*. [40]. Prior to the analysis, the protein samples were acetylated *in vitro *using sulfo-N-hydroxysuccinimide acetate (Pierce, Rockford, IL). The samples were then subjected to thermal treatment for 20 min in the presence of SDS and 2-mercaptoethanol. After the treatment, the oligomeric states of the proteins were assessed using SDS-PAGE and Coomassie Brilliant Blue (Serva, Heidelberg, Germany) staining.

### Limited proteolysis with proteinase K

The stability of xenavidin against the proteinase K enzyme, which is known to cleave the L3,4-loop of avidin [41], was tested. The assay was performed in the presence or absence of biotin as previously described by Laitinen *et al*. [64]. Protein samples were taken before the enzyme treatment and after adding enzyme at time points 30 min, 1 h, 2 h, 3 h, 18 h and 22 h (data not shown), and the samples were analyzed using SDS-PAGE analysis and Coomassie Brilliant Blue (Serva, Heidelberg, Germany) staining.

### Immunological cross reactivity

The cross-reactivity of anti-streptavidin or anti-avidin polyclonal antibodies was tested with xenavidin using sera samples that where gathered from cancer patients who were either exposed to streptavidin or avidin (positive samples) or neither of the proteins (negative samples). The serum samples were kindly provided by Professor Giovanni Paganelli from the Division of Nuclear Medicine, European Institute of Oncology, Milan, Italy. The cross-reactivity of polyclonal rabbit antibodies produced against avidin (University of Oulu, Finland) and streptavidin (a generous gift from Edward A. Bayer, Weizmann Institute of Science, Jerusalem, Israel) was also analyzed with xenavidin. All of these experiments were conducted using ELISA, as previously described in detail [13].

### Structure-based sequence alignment

The Cα atoms (A subunits only) of the crystal structures of avidin [PDB: 1AVD], streptavidin [PDB: 1MK5] and xenavidin (reported here; [PDB: 2UYW]) were structurally aligned using the inbuilt tools of Pymol [65]. The resulting ensemble of aligned structures was downloaded into Bodil [66] to create the structure-based sequence alignment *per se*; residues that had Cα atoms close to each other (visual checking) were edited to be matched. A graphical presentation of the structure-based sequence alignment was drawn with ESPript 2.2 [67] using the default similarity scores (global score 0.7 and diff. score 0.5). Some amino acid residues in the structures of avidin, streptavidin and xenavidin had more than one atom in close proximity (< 4.5 Å) with biotin and, hence, more than one type of interaction (e.g. hydrogen bonding and hydrophobic effect) with biotin was theoretically possible. However, only one type of interaction for each residue is shown in the alignment (Figure 1); hydrogen bonds are preferentially indicated.

### Miscellaneous methods

The electropotential calculations (Figure 2) were done using the ABPS plugin of Pymol [68] and if more than one rotamer were found to be built for amino acid residues, only the A conformers were used. Figure 1A, Figure 2 and Figure 3 were created with the PyMol Molecular Graphics System [65] and CorelDRAW 11 was used to edit these figures. Figure 4 and Figure 5 were scanned with a scanner and edited with Adobe Photoshop CS.

## Authors' contributions

The production of xenavidin and biochemical studies of xenavidin, avidin and streptavidin were carried out in the group of MSK at the University of Tampere - Institute of Medical Technology. The structural analysis was done in the group of MSJ at the Structural Bioinformatics Laboratory, Åbo Akademi University. JAEM performed the protein expression and most of the protein biochemical analyses, SHH designed and carried out immunological cross reactivity assay, VPH was involved in the ligand-binding analyses. TTA designed and carried out the 3D-structure analysis of xenavidin. HRN supervised the experimental work related to biochemical analyses. JAEM, VPH, MSK, TTA and MSJ were mainly responsible for writing and editing the manuscript. All authors read and approved the final manuscript.

## Supplementary Material

Additional file 1 **Oligomeric state of xenavidin**. Gel filtration analysis of xenavidin. Click here for file

Additional file 2 **Oligomeric state of xenavidin-biotin complex**. Gel filtration analysis of xenavidin with biotin ligand. Click here for file

Additional file 3 **Immunological cross reactivity**. The immunogenic reactivity of sera samples collected from cancer patients exposed to either avidin or streptavidin (or both) were tested against xenavidin, avidin and streptavidin. Click here for file

Additional file 4 **Fluorescence emission spectrum of Bf560-biotin**. The fluorescence spectrum of Bf560-biotin conjugate in the presence and absence of chicken avidin. Click here for file
