# Supplementary material for: Structural and functional characteristics of xenavidin, the first frog avidin from Xenopus tropicalis
Source: BMC Struct Biol. 2009 Sep 29;9:63. doi: 10.1186/1472-6807-9-63 (PMC2761383; doi:10.1186/1472-6807-9-63)
Supplement: Additional file 1 — Oligomeric state of xenavidin. Gel filtration analysis of xenavidin. [file 1472-6807-9-63-S1.DOC]

# Additional files


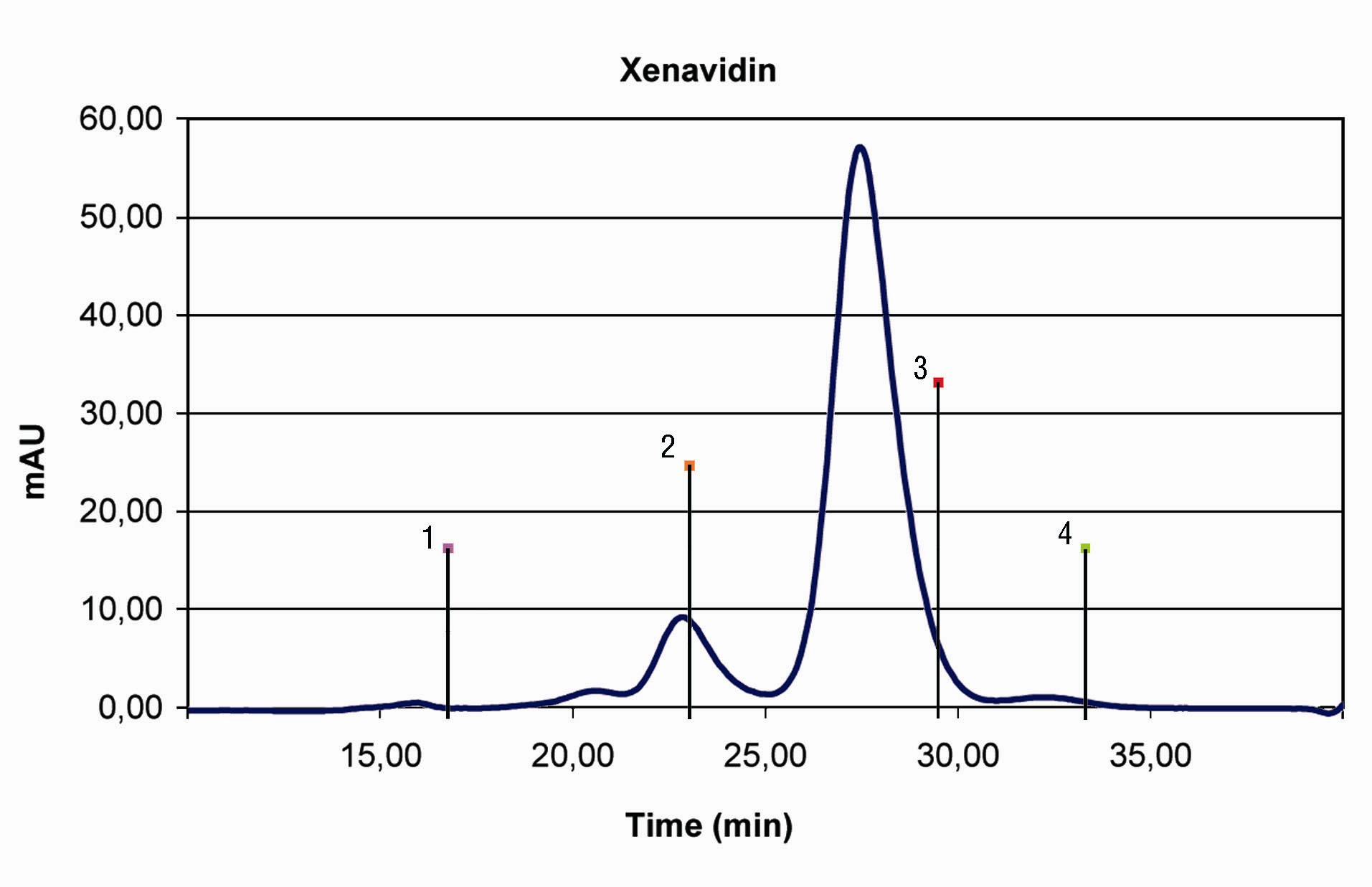


### 45BAdditional file 1 – Oligomeric state of xenavidin

The samples were analyzed using an ÄKTA^TM^ purifier HPLC instrument (Amersham Biosciences) equipped with a Superdex 200 10/300 GL column (Tricorn) (see text for details). The absorbance (mAU) at wavelength of 280 nm plotted against time (min) is shown. The elution times of gel filtration standard proteins (Bio-Rad) are indicated by numbered labels as follows: (1) thyroglobulin (670 000 Da), (2) γ-globulin (158 000 Da), (3) ovalbumin (44 000 Da) and (4) myoglobin (17 000 Da).
