# Supplementary material for: Structural and functional characteristics of xenavidin, the first frog avidin from Xenopus tropicalis
Source: BMC Struct Biol. 2009 Sep 29;9:63. doi: 10.1186/1472-6807-9-63 (PMC2761383; doi:10.1186/1472-6807-9-63)
Supplement: Additional file 3 — Immunological cross reactivity. The immunogenic reactivity of sera samples collected from cancer patients exposed to either avidin or streptavidin (or both) were tested against xenavidin, avidin and streptavidin. [file 1472-6807-9-63-S3.DOC]

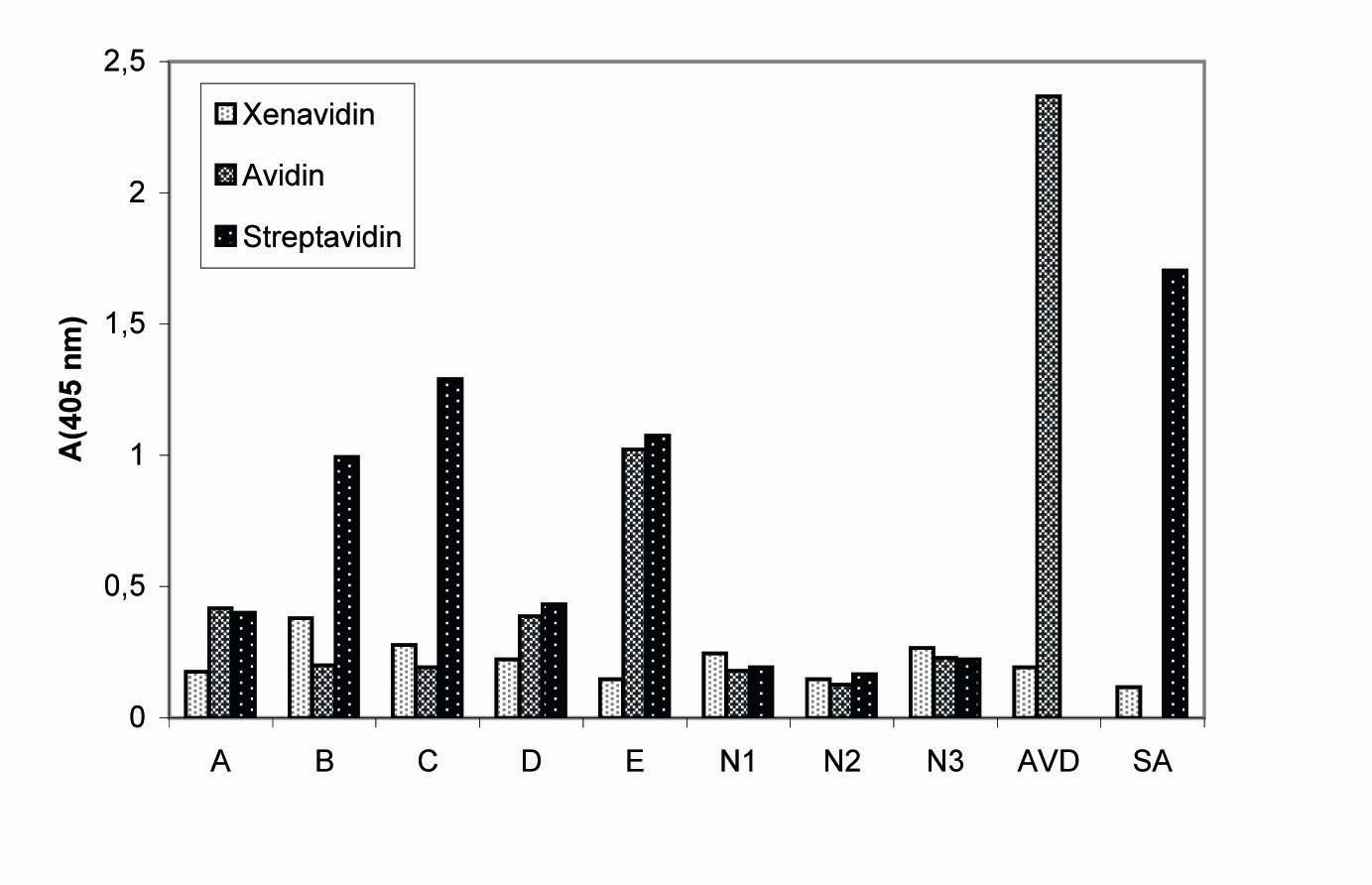


### Additional file 3 – Immunological cross reactivity

Sera samples, labeled A through E, were gathered from cancer patients exposed to either avidin or streptavidin (or both), and the reactivity against xenavidin, avidin and streptavidin were tested. Negative control sera, N1 through N3, were from controls not exposed to avidin or streptavidin. Cross-reactivity of polyclonal rabbit antibodies against avidin (AVD) and streptavidin (SA) were also tested.
