# Supplementary material for: Structural and functional characteristics of xenavidin, the first frog avidin from Xenopus tropicalis
Source: BMC Struct Biol. 2009 Sep 29;9:63. doi: 10.1186/1472-6807-9-63 (PMC2761383; doi:10.1186/1472-6807-9-63)
Supplement: Additional file 4 — Fluorescence emission spectrum of Bf560-biotin. The fluorescence spectrum of Bf560-biotin conjugate in the presence and absence of chicken avidin. [file 1472-6807-9-63-S4.DOC]

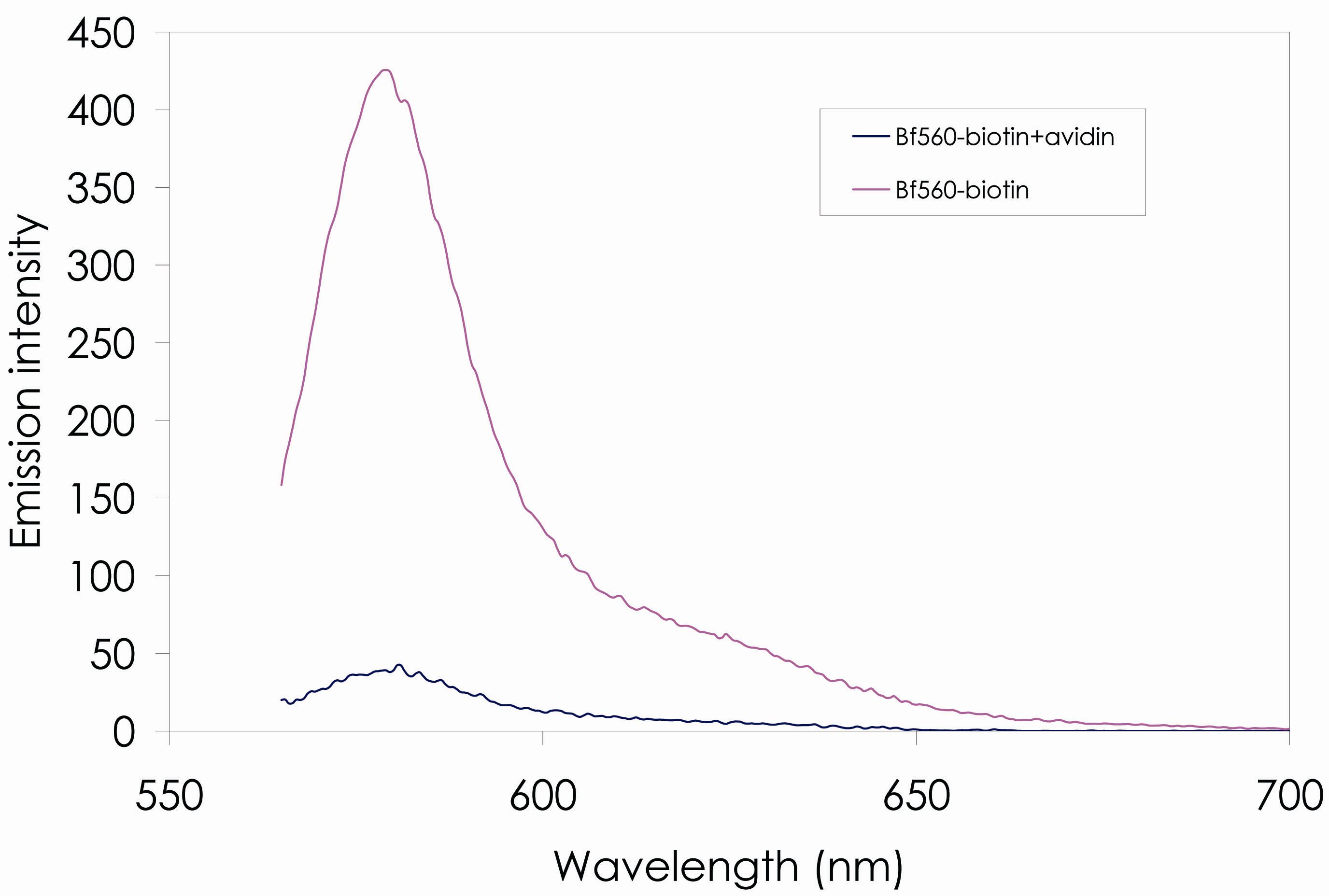


**Additional file 4 – Fluorescence emission spectrum of Bf560-biotin**

Emission spectrum of Bf560-biotin in the presence and absence of avidin.
